# Potential Future Directions in Optimization of Students' Performance Prediction System

**DOI:** 10.1155/2022/6864955

**Published:** 2022-05-17

**Authors:** Sadique Ahmad, Mohammed A. El-Affendi, M. Shahid Anwar, Rizwan Iqbal

**Affiliations:** ^1^EIAS: Data Science and Blockchain Laboratory, College of Computer and Information Sciences, Prince Sultan University, Riyadh 11586, Saudi Arabia; ^2^Department of Artifitial Intelligence and Software, Gachon University, Seongnam, Republic of Korea; ^3^Department of Computer Engineering, Bahria University, Karachi Campus, Karachi, Pakistan

## Abstract

Previous studies widely report the optimization of performance predictions to highlight at-risk students and advance the achievement of excellent students. They also have contributions that overlap different fields of research. On the one hand, they have insightful psychological studies, data mining discoveries, and data analysis findings. On the other hand, they produce a variety of performance prediction approaches to assess students' performance during cognitive tasks. However, the synchronization between these studies is still a black box that increases prediction systems' dependency on real-world datasets. It also delays the mathematical modeling of students' emotional attributes. This review paper performs an insightful analysis and thorough literature-based survey to draw a comprehensive picture of potential challenges and prior contributions. The review consists of 1497 publications from 1990 to 2022 (32 years), which reported various opportunities for future performance prediction researchers. First, it evaluates psychological studies, data analysis results, and data mining findings to provide a general picture of the statistical association among students' performance and various influential factors. Second, it critically evaluates new students' performance prediction techniques, modifications in existing techniques, and comprehensive studies based on the comparative analysis. Lastly, future directions and potential pilot projects based on the assumption-based dataset are highlighted to optimize the existing performance prediction systems.

## 1. Introduction

Over the past few decades, students' performance has been predicted while evaluating the influence of different factors, such as emotional attributes, family attributes, study schedule, institutional attributes, and students' scores in assignments, quizzes, and final examinations [1–5]. Such systems provide useful applications to a wide area in academia, i.e., students' success and failure estimation due to influential factors [6–10]. This study splits the earlier contributions into two groups. The first group consists of insightful psychological studies, data mining discoveries, and data analysis findings that indirectly contribute to the optimization of students' performance prediction systems. The second group reports the optimization of existing prediction systems based on the findings of the first group. However, the extensive synchronization between the two groups is still a black box that ultimately increases students' performance prediction systems' dependency on a real-world dataset. Such synchronization can provide useful ideas during the optimization and data collection process. It also paves the way for an assumption-based dataset to prove the viability of pilot project implementations that will speed up modeling students' emotional attributes.

This review paper conducts an insightful study and literature-based survey to draw a comprehensive picture of the prior studies on student performance analysis and prediction. The review consists of 1497 articles from 1990 to 2022 (32 years), which reported various information for future researchers:

1. It explores and lists the research fields' contributions focusing on students' performance optimization. Psychology and data analysis fields pave the way for effective solutions to the problems of data deficiency. They provide qualitative findings that can be used for creating an assumption-based dataset for pilot project implementation.
2. It thoroughly considered new and modified algorithms that predict students' performance. Also, a comparative analysis was performed between the existing students' performance prediction approaches to provide better recommendations for optimization.
3. The study delivers a comprehensive picture of potential challenges and research direction for future researchers. The review also shows that very few contributions have mathematically modeled emotional attributes.

The remaining sections of this review are as follows: Section 2 gives a detailed literature review.  Section 3 elaborates the review methodology, and Section 4 produces data evaluation.  Section 5 presents future challenges, and Section 6 concludes the study.

## 2. Literature Review

Students' performance prediction systems have enormous applications in academia, such as predicting at-risk students, course recommendation, and basic counseling against negative emotions, highlighting the influence of institutional attributes, family factors, etc. [11–14]. It is also needed to advance the academic achievement of excellent students [15–19]. Prior studies deliver qualitative and quantitative results in extending students' performance evaluation and calculation, and highlighting the factors that influence the performance [20–25]. For a few decades, psychology, data mining, cognitive computing, and data analysis fields directly or indirectly contributed to the optimization of students' performance prediction systems [26–34]. Therefore, related work is split into the following subsections.

### 2.1. Contributions of Psychology

Psychological studies results manifest that students' performance is easily influenced via emotional attributes, such as frustration, anxiety, stress, over expectation of parents, and parents' relationship [35–37]. The results provide correlations statistics among emotional factors and the expected performance of students in cognitive activity, such as attempting the examination, quizzes, assignments, class activities, and extracurricular activities. These particular emotional factors can negatively and positively impact the students' performance. In such a situation, emotional severity, family attributes, and institutional factors play a crucial role in influencing performance [38–40]. It shows that performance is always very sensitive and affected by the individuals' surroundings.

### 2.2. Contributions of Data Mining

The data mining evaluates the relationship among various students' factors, such as the role of emotional factors, family attributes, institutional factors, and class performance. Such studies provide good opportunities for accurate estimations of expected students' performance [41–47]. The meaningful patterns always produce good directions for further exploration. The previous articles lack coordination between the students' influential attributes and academic performance. The literature lacks accurate techniques to simulate students' performance due to the insufficient synchronization and coordination among earlier studies on students' factors. The mathematical modulation of students' performance needs to formulate the function of student factors. However, it is inspiring to closely examine the quantitative influence of several student factors on academic achievements. The earlier studies show that emotional, family, study schedule, and institutional attributes are the significant factors that can easily influence students' academic performance in any critical cognitive activity. Prior studies illustrate that educational data mining practices contribute to students' factor evaluation process and performance prediction. Institutional factors involve teaching methodology, engaging students in the classroom, and the vision of instruction. According to literature studies, teachers play an active role in institutional attributes influencing students' performance. They provide administrative assistance and assistance in ensuring discipline [48–57].

### 2.3. Synchronization among Existing Studies

Accurate performance prediction needs to examine students' factors beyond the computer science framework. The literature studies are still limited in finding an authentic and extendable approach that overlaps psychology, data mining, data analysis, and cognitive research. Articles have various solutions to predict students' performance using different techniques that could have the potential to be escalated to more general problems of predicting student performance [58–62]. The primary objective of the current review attempt is to efficiently explore the relationship between students' factors (as mentioned earlier) and their performance. Therefore, the literature is studied with the selected students' attributes (emotional, family, study schedule, and institutional) and effects. Articles show that most students do not participate in extracurricular activities, believing extra activities would negatively affect their academic achievements. Earlier studies also focus on predicting college students' performance by considering all the important aspects. They delivered a prediction system to estimate performance by assisting the university in selecting each candidate using past academic records of students granted admissions [29, 63–69]. Such efforts show that earlier studies contribute to decreasing the number of at-risk students and advancing the performance of excellent students.

Literature also attempted to perform a survey on classroom learning in different environments. It analyzes various aspects and factors influencing (positively or negatively) performance in a classroom that interfere with learning. This paper presents a systematic review of numerous studies on students' performance in classroom learning. For a few decades, the research has produced numerous results in students' performance evaluation; however, the education system needs a complete and detailed performance prediction system that can ensure interaction and coordination between the aforementioned students' factors. Literature studies delivered various contributions, such as the proposal of an innovative model that targets modifying learning sustainability through smart education applications and regression and correlation among students' factors, and logistic regression analyses generated that being female, first-semester GPA, number of courses per regular semester, and number of courses per summer semester were imperative predictors of baccalaureate degree achievement [70–77].

### 2.4. Existing Models and Performance Prediction System Optimization

Studies have focused on applying artificial neural networks to predict performance in different environments. Articles are also saturated with deep learning techniques that deliver prediction and highlight at-risk students. Few other technologies provide opportunities to accurately evaluate the performance and reduce the failure rates [78–82]. It also helps in counseling students in alarming situations that can positively impact their academic achievements, i.e., COVID-19. Thus, during the literature survey, we have found many students' prediction systems which are interesting; nevertheless, they are failed to mathematical model emotional attributes and synchronized them with institutional attributes, study schedules, and family attributes [31, 37, 83–98]. The objective of this study is to identify the relationship between extracurricular activities and students' performances.

The articles deliver many results on the effects of influential students' factors. This study explores performance prediction beyond the scope of computer science and machine learning.

### 2.5. Related Performance Prediction Methods

As discussed earlier, many studies solved meaningful challenges in students' performance prediction area of research for a few decades. The earlier studies have many contributions in the form of neural works, recommendation systems, course recommendations, and students' performance evaluation systems [87, 99–109]. The prior studies demonstrate comprehensive work on students' performance prediction systems that use information obtained during the interaction of students with the institutional attributes. To mathematically consider the expected actions of a students' factors, such information provides proper guidelines. The significant characteristic is the identical structure of the information processing system of students, which can be replicated to construct a learning algorithm (cognitive architecture). Literature studies are flooded with many findings that primarily contribute to prediction algorithms and mathematical models; nevertheless, modeling the relationship between students' emotions (frustration, stress, etc.) and students' performance is very little focused [110–115].

Also, the published studies on modeling emotion are not extendable toward a matured prediction system. So, the dire need is to assess the main framework of existing prediction algorithms. Exploring the qualitative results of psychological studies and data analysis discoveries is needed to estimate students' performance. It will also help in the iterative calculation of emotional influence on performance during critical cognitive activities.

Extraordinary academic performance is only possible with excellent cognitive skills. Such skills are needed to accomplish any task requiring problem-solving approaches, reasoning, and memory management. However, with inadequate cognitive abilities, an individual cannot achieve an excellent score in various cognitive tasks, i.e., assignments, quizzes, and written examinations. They require students to process new information, organize learning, and retrieve that data (from memory) for later use. So, predicting performance while calculating the intense impact of various groups of factors is crucial not only for tutors to ensure effective teaching methodology but also for students' achievements and effective academic policies. Earlier studies have delivered many approaches that predict students' performance; nevertheless, they have paved the way for new challenges for effective educational systems. The skills levels of students are changing as they learn and forget. The educational system needs such a system that can manage the students' dynamic behavior during cognitive activities.

Other studies have described the students' personality traits and the essential characteristics of personality. Results reveal that performance prediction design can be broken down into subsections that are more realistic in comparison to other techniques. It also paves the way for the development of performance prediction architectures which were easy to understand. The current review illustrates that the performance prediction system needs to coordinate among prediction architecture, psychological experiments, semantical investigations, statistical analysis, and mathematical formulation. This work provided an outstanding opportunity for researchers belonging to performance prediction, bioinformatics, data mining, and data integration.

## 3. Review Methodology

**Figure 1:**
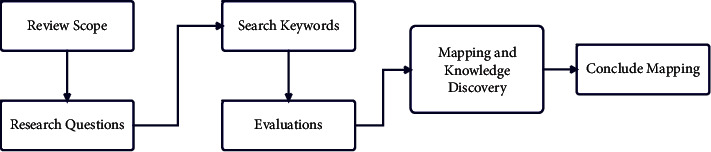
Framework illustrated the main modules of the current review process.

The review process is started from the initial screening within the scope of the current attempt. As elaborated earlier, this review focused on recent and state-of-the-art contributions to student performance prediction.  Figure 1 illustrates the methodology of the review. We have divided the complete review process into the following sections.

### 3.1. Review Process

This study reviews the earlier studies thoroughly based on the procedures prescribed by Petersen et al. [116] and Keele [117]. The methodology is adopted from Keele while the study mapping method is copied from Petersen et al. The review process is initiated with the modified procedure, which is demonstrated in Figure 1. For better understanding, the review delivers a detailed methodology of the prior work contributing to student performance prediction directly or indirectly. Moreover, the study put a list of research questions to demonstrate the main objectives. These research questions enable us to choose relevant research studies for screening and investigating the main challenges in students' performance predictions. Every research question has a list of keywords to explore the literature and learn about a particular question. These keywords are used to search publications, including peer-reviewed book chapters, conferences proceeding, and journal articles.

### 3.2. Research Questions

1. Q1: what are the applications of student performance prediction systems?
2. Q2: what are the factors that can optimize student performance prediction?
3. Q3: what is the intensity of research findings in the field of student performance prediction systems optimization?
4. Q4: are the findings of psychological studies, data mining, and contribution in algorithms synchronized with each other for the viability of the pilot project?
5. Q5: how synchronization and coordination of prior psychological, data mining, and algorithmic findings contribute to the effective educational system via student performance prediction algorithm.

### 3.3. Searching Keywords

The current study adopted a manual review methodology introduced by Keele [117]. The automatic review presented by Petersen et al. has a few disadvantages [116]. (1) The automatic search is not feasible for the current review [118]. (2) The manual searching strategy gives more relevant studies.  Table 1 reflects the list of keywords that have produced a variety of articles published by various publishers, i.e., IEEE, Elsevier, Springer, Hindawi, MDPI, ACM, Wiley, and others. It shows many articles, including journals, book chapters, and conference proceedings.

The keywords were searched directly on publishers' websites and Google Scholar with a default setting. We have evaluated all the articles and collected those that deliver relevant findings for further screening. Furthermore, the main factors, topics, and relevant studies, including journals, conference proceedings, and book chapters, are given below:

1. Emotional attributes
2. Family factors
3. Study Schedule
4. Institutional attributes
5. Psychology, data mining, and data analysis findings on the factors as mentioned earlier
6. Contribution of cognitive computing, deep learning, and machine learning in students' performance prediction
7. Reviews and comparison

### 3.4. Screening

Screening of studies is performed with the following terms and conditions:

1. The team selected the publications of the more relevant journal, conference, and book chapter.
2. Second, we have focused on the relevant title with impressive citations in Google Scholar.
3. Third, rapid reviews were performed for further evaluation and data extraction. During the rapid review, we have focused on the abstract and introduction to get some idea about the challenges, motivations, and contributions.

These three steps were performed to create a database for further information extraction and data collection.

### 3.5. Information Collection

Various information was extracted from the selected publications during the information collection process, which are shown in Table 1to 4 Also, a spreadsheet was used to record the various information for further consideration of the research questions. The recorded data are shown in the tables mentioned above.

## 4. In-Dept Analysis

### 4.1. Q1: What Are the Applications of Student Performance Prediction Systems

A performance prediction system is essential to predict at-risk students to devise a solution for successful graduation and goal achievements, such as special treatment and counseling sessions. Such prediction systems are more challenging due to the significant factors affecting students' performance. Thus, a systematic review of the literature has been performed to highlight potential issues in predicting student performance. The study also shows the contributions of previous articles beyond the scope of artificial intelligence, i.e., data mining, data analysis, and psychology techniques contributing to performance prediction. Also, this study provides an overview of prediction techniques that have been used to estimate performance. It focuses on how the predictive algorithm can be used to identify key attributes in influencing students' academic achievements. With the help of data mining and machine learning techniques in education, the study could have a more effective methodology in proposing a new prediction algorithm and modifying existing students' performance prediction systems. The primary application outcomes of students' performance prediction are given below.

#### 4.1.1. Prediction of At-Risk Student

It is crucial to predict at-risk students and devise an effective learning environment in classrooms and laboratories. Although the literature studies are saturated with tremendous results, it is still challenging as the prediction system cannot synchronize and mathematically model emotional attributes, family issues, study schedules, and institutional attributes to develop a significant prediction system. The current review's first target is to highlight the possibilities of predicting at-risk students while coordinating between literature studies.

#### 4.1.2. Advances the Students' Academic Achievements

The performance prediction system is essential for at-risk, average, and excellent students. The influential factors that drive academic achievement are an eternal global challenge associated with students, families, teachers, and educational policymakers. Exploring these factors benefits all those interested in developing a system for students' performance prediction worldwide. Suppose the prediction system considers a large number of influential factors. In that case, the academic achievement of excellent students can also be advanced, i.e., the prediction system could highlight problems due to various emotional, study schedules, family, and institute-related attributes.

#### 4.1.3. Monitoring Students' Behavior

Student behavior plays a significant role in improving academic achievements, such as interaction and attitude with the teachers, seriousness, and unseriousness in the classroom. Articles of psychology and data analysis contribute to student behavior evaluation, merits, and demerits of various aspects of behavior. We need a prediction system that efficiently modulates the relationship between behavior and students' performance to highlight, monitor, and improve students' interaction and engagement in the classroom. It is also essential for the institution to devise effective controlling policies to counter and control the demerit of various behaviors. Through such a prediction system, teachers can easily guide their students in setting and achieving academic goals. A teacher can also help students understand their behavior and its impact on others. The adverse effects of behavior can be overcome and later on monitored by supervising students. Such a system enhances the overall reputation of the institution. Other benefits include preventing early school drop-ups and building good relationships among students. According to Kennelly and Monrad [119, 120], the behavioral problem plays a key role in indicating students at risk and highlighting the individuals near to being dropped off at the institute. Therefore, employing strategies to monitor and control student behavior is extremely important for an effective educational system in a society.

### 4.2. Q2: What Are the Factors That Can Optimize Student Performance Prediction?

The literature studies indicate that many factors influence students' performance in cognitive activities, such as quizzes, assignments, examinations, and homework. It includes family-related factors, emotional factors, gender description, and institution-related factors. A brief description of these factors is given below.

#### 4.2.1. Family-Related Factors

The parental involvement and their particular influence are two-fold. First, the earlier studies claim that the interaction of parents positively influences performance. It enhances the academic achievements of the student in critical environments. Research results highlight that parents' friendly attitudes positively affect student performance, such as daily engagement in cognitive activities. Positive parent involvement can advance the performance, and that father or mother is the first teacher who plays the role of an enduring educator. Such research findings show that parents' positive and active role cannot be underestimated. Second, the overexpectation of parents can push children towards frustration [121, 122]. Parent mostly observes remarkable achievement on social media, so they also start demanding good grades from their children. With such pressure, students are easily frustrated, which negatively influences their academic outcome during cognitive activities, such as assignments, quizzes, and mid- and final-term examinations. So, the role of the parents should be supportive and motivational, which would help against unnecessary pressure.

To achieve a student performance prediction system, we need to consider parental involvement and the aforementioned other attributes, such as the cohabitation status of parents, the relationship among their parents, socioeconomic situation, and the number of children. Prediction systems need to quantize all these attributes to evaluate future student performance properly. If we look into literature studies, a minimal contribution can be evidenced toward mathematical modeling of student performance for a better educational system.

#### 4.2.2. Emotional Factors

Emotional attributes play a fundamental role in impacting student performance during cognitive tasks. The current study discusses severity levels of frustration, anxiety, depression, and stress. The impact of frustration is the natural part of learning as well as the engaging session (for references, see the literature review section). Such emotion is always found during comprehensive cognitive activities. Literature saturated with many qualitative findings focused on the statistical association between student performance and frustration. However, the study has not been evidenced a comprehensive approach to solve the challenges produced by frustration during cognitive tasks.

We need to analyze the performance of excellent, average, and at-risk students while mathematically modeling the relationship between institution-related attributes, students' emotional factors, and family-related attributes. Also, the teacher can help frustrated students' through collaborative exercises, group activities, and group assignments [123]. It will help students easily share their confusion and problems with group members to overcome their frustrations in a comprehensive learning environment. An individual can learn better in offline mode with face-to-face interaction as compared to online interaction [124]. Additionally, the COVID-19 outbreak has accelerated the influence of negative emotions on students' performance. COVID-19 has created a more critical situation for students' learning and adjusted them to the online environment with fewer resources. Thus, we are in dire need to evaluate the academic development of students while statistically associating the aforementioned factors and mathematically modeling the proposed relationship to prepare for the critical situation [125].

#### 4.2.3. Gender Description

In the literature review section, the study has shown that earlier studies statistically associated students' performance with emotional attributes and gender description. Students perform differently while considering aging and gender [126]. Both emotion and gender need to evaluate differently during cognitive activities. Literature studies are evidenced with many contributions on gender differences. They show that different gender individuals perform differently during cognitive activities, solving assignments, attempting quizzes, and examinations. Earlier studies depict that gender difference is an independent biological factor whose magnitude is sometimes dependent on other factors such as cultures, socioeconomic condition, language, age, etc. Gender differences play a crucial role in influencing mental abilities and cognitive processing in mathematical tasks, physics, research, reading, and writing. These issues create a big gap between male and female individuals, referred to as natural and biological differences.

#### 4.2.4. Institution-Related Factors

Different institutional factors are directly or indirectly involved in influencing students' performance. These factors include but are not limited to instructor teaching methodology, interaction with a student advisor, extracurricular activities in the institution, student complaint platform, the distance between the institution and students' residence, transport facility, and the behaviors of the friends. These all factors have merits and demerits for student performance. The literature studies of psychology and data analysis have enormous contributions to student performance analysis; however, insignificant contributions have been reported in the form of algorithms and mathematical models in students' performance prediction.

### 4.3. Q3: What Is the Intensity of Research Findings in the field of Student Performance Prediction Systems Optimization?

Literature reported many challenges because the students' performance prediction overlaps psychology, data analysis, and mathematical and algorithmic contributions. The intensity of publications in the student performance prediction area is reported below.

#### 4.3.1. Intensity of Psychological Findings

As discussed earlier, we can find many psychological research contributions in the field of student performance analysis, which show that emotional attributes always affect students' performance during cognitive activities. So, to provide an efficient solution for student performance prediction, the study must need to evaluate the psychological findings that directly or indirectly focus on student performance evaluation.

#### 4.3.2. Intensity of Data Analysis Findings

Data analysis contribution provides a quantitative measurement for student performance prediction. Such research findings pave the way for an accurate mathematical model to better contribute to the performance prediction area of research.

#### 4.3.3. Intensity of Students' Performance Prediction Systems

The literature is also saturated with student performance prediction techniques focusing on students' performance prediction in critical cognitive tasks; nevertheless, these findings are not synchronized and linked toward a significant student performance model. So, the main objective of this review paper is to provide an effective platform for future researchers in student performance prediction. It will pave the way for an effective system to predict at-risk students and excellent student performances, which ultimately provides us with the opportunity to enhance their skills and performance.

### 4.4. Q4: Are the Findings of Psychological Studies, Data Mining, and Contribution in Algorithms Are Synchronized with Each Other for the Viability of Pilot Project?

The intensity of publications contributing to student performance prediction is quite good, but these contributions are not synchronized with each other to mathematically model emotional, family, and institution-related attributes. One of the main objectives of the current review is to highlight the lack of coordination and synchronization of the literature from different research fields. This review would allow future readers of deep learning to collaborate with other research fields.

### 4.5. Q5: How Synchronization and Coordination of Prior Psychological, Data Mining, and Algorithmic Findings Contribute to the Effective Educational System via Student Performance Prediction Algorithm

Psychological literature produces both qualitative and quantitative findings in students' performance prediction; nevertheless, the data analysis field highlights the association among students' factors, i.e., emotional, family, and institutional attributes. If these findings are linked with the objective of qualitative data repositories and algorithms, then, we can move toward an efficient student performance prediction system. The psychological work produces accurate students' emotional data focusing on their performance. On the other hand, the data analysis field makes the meaningful statistical association and correlation information. The data analysis field of research provides a couple of tests to find the correlation between student emotional attributes and their performance, i.e., Pearson correlation and regression. These tests verify the correlation among different factors.

We are in dire need to have the abovementioned psychological and data analysis findings to propose a comprehensive algorithm. Every part of the student performance prediction area of research is interlinked. The psychological result verifies the emotional change during the evaluations of the frustration, severity, anxiety, and stress. Second, the data analysis findings associate the student attributes. Third, the student performance prediction algorithm mathematically model the statistical association among the student influencing factors and their performance outcome.

### 4.6. Specific Keywords-Wise Publications

This section intensively discusses the specific keyword-wise research output focusing on students' performance, emotional factors, and prediction algorithms. The list of keywords is illustrated in Table 1. The study collected articles based on these keywords for further technical assessment. The specific domain for the technical evaluation includes but is not limited to new methods, modifications in prior work, data analysis, psychological findings, application analysis, review work, and comparison. The self-explanatory Table 1 illustrated the intensity of publications in the domain above using the list of keywords.

### 4.7. Yearly Publications

**Figure 2:**
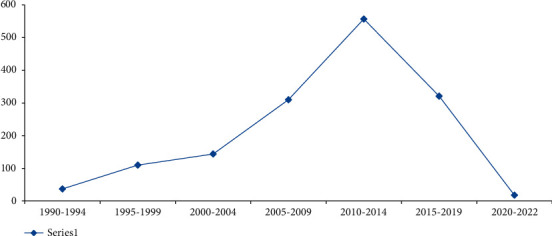
Yearly research contributions.

Literature studies deliver thousand of research findings that directly or indirectly contribute to students' performance analysis and prediction. As illustrated in Figure 2, 37 published articles were evaluated (1990 to 1994). About 110 articles mainly focus on student performance and students' study-related factors assessment. They have evaluated those factors that affect students' performance during cognitive activities (1995 to 1999). From 2000 to 2004, the study included 144 articles on students' performance and emotional attributes. The number of featured articles increases with time. From 2005 to 2009, we have assessed 310 articles that contributed to performance prediction.

Furthermore, we have collected 557 research studies that mainly focused on prediction algorithms. The researchers delivered a considerable amount articles from 2010 to 2014. We have found a slight decrease in analysis and psychological studies production until 2019; however, an increase was observed in algorithmic work from 2015 to 2019. Thus, 321 studies were considered during this segment of time. Eventually, the study reviewed 18 articles on students' performance prediction algorithms published from 2020 to 2022. We have collected 1497 articles during the review process of the current study.

### 4.8. Domain-Wise Evaluation

**Figure 3:**
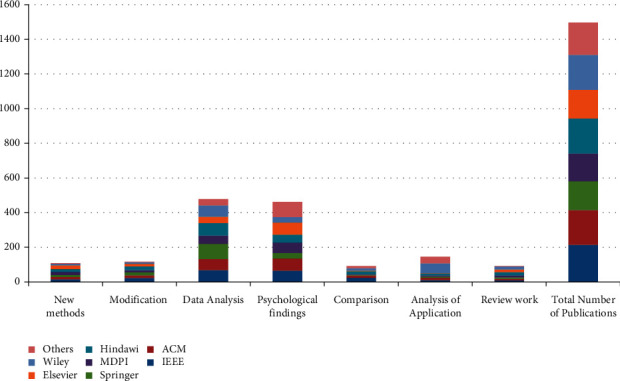
Domain-wise and publishers-wise outcomes.

**Figure 4:**
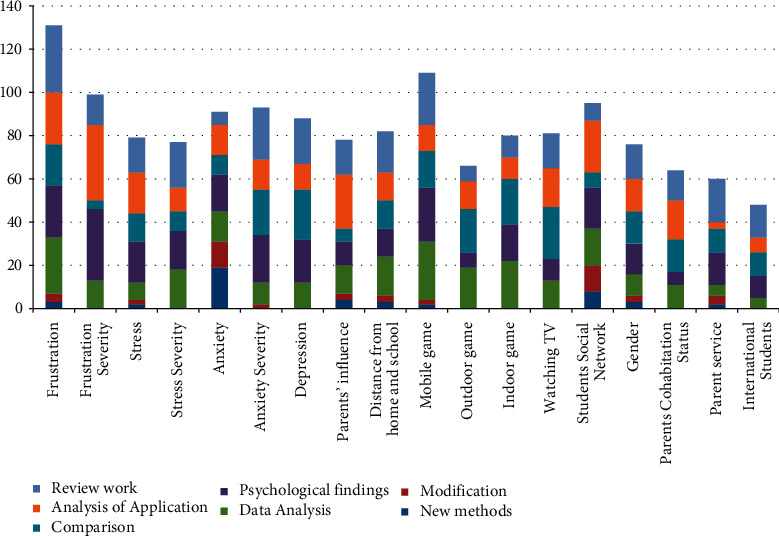
Factors-wise and domain-wise outcomes.

The review paper carries out a perceptive analysis and literature-based survey to draw out an inclusive representation of the famous publishers who publish the most highly cited research papers. It involves overall 1497 publications, which are selected after searching on Google Scholar. These studies were evaluated upon their relevant findings, such as new methods, modified approaches, statistical findings, and psychological results (for more information see Table 2 and Figure 3). The major part of this review was to assess the existing students' performance prediction approaches. So, the study analytically assessed new students' performance prediction measures, modifications in state-of-the-art techniques, and comparative analysis. Additionally, Figure 4 and Table 3 represent the detailed domain-wise and factors-wise analysis.

## 5. Potential Future Challenges

A large number of factors are involved in influencing students' performance; therefore, the prediction system needs to be optimized to consider the impacts of different human factors categories. Such factors categories include but are not limited to emotional attributes, study schedule, family attributes, and institutional attributes. Each category consists of multiple factors impacting students' performance, either negatively or positively. In the literature section, the study provides a detailed discussion of these factors.

### 5.1. Potential Pilot Projects Based on the Assumption-Based Dataset

The comprehensive synchronization between the earlier studies is still a black box, which increases systems' dependency on a real-world dataset. The importance of a real-world dataset cannot be avoided; however, the data collection process is time-consuming and need a list of human resources. It delays the optimization of existing approaches, such as modeling students' emotional attributes. The data collection process could have various anomalies if the researcher does not follow the analysis of earlier studies. The earlier studies offer excellent opportunities to understand the effectiveness of emotional attributes for optimization. Therefore, pilot projects perform key roles in optimizing the existing students' performance prediction systems. They provide useful ideas during the data collection process. They also pave the way for an assumption-based dataset to prove the viability of novel ideas in students' performance prediction.

## 6. Additional Points of Earlier Studies

Data analysis findings explore the hidden patterns and statistical correlation between students' performance and influential factors. Such opportunities introduce new challenges for students' performance prediction systems, e.g., conditional probabilities, correlation, and inferencing. Also, data mining studies are evidenced with many findings in students' performance prediction area of research; nevertheless, they have different limitations, e.g., lack of in-depth investigation of students' performance based on selected study-related factors, limited scalabilities, limited dataset, and inadequate qualitative approach of data analysis and psychological studies.

Finally, the review shows that various prior students' performance prediction methods have been proposed in the last decade; however, meagre studies have highlighted the basic need for synchronization among the abovementioned field's contributions. Therefore, this review provides an exclusive picture of the future challenges in students' performance prediction (see Table 1to 4 and Figure 2to 4). On one hand, Table 4 depicts the intensity of various optimization techniques, review works, and new students' performance prediction methods. On the other hand, Table 5 represents the acronyms of the selected studies. Remarks and recommendations against each research question are given in the self-explanatory Table 6.

## 7. Conclusions

The proposed review highlights the potential research opportunities to optimize the students' performance prediction systems while exploring earlier contributions of different research fields, i.e., cognitive computing, data mining, data analysis, and psychology. The previous studies are still limited in synchronization between the existing contributions of various fields, which negatively impacted the mathematical modeling of emotional attributes. It increased the systems' dependencies on real-world datasets. Thus, to investigate the potential challenges thoroughly, the study is split into three sections.

1. The data mining discoveries, psychological findings, and data analysis results are examined.
2. The study performs a domain-wise investigation of the existing methods focusing on students' performance prediction, i.e., the domain includes new students' performance prediction techniques, modifications in existing techniques, and comparisons analysis.
3. Eventually, future direction and potential pilot project viability are highlighted.

## Figures and Tables

**Table 1:** Result obtained from the Google Scholar during keyword searching.

No	Keywords	IEEE	ACM	Springer	MDPI	Hindawi	Elsevier	Wiley	Others	Total
1	Student performance prediction	14	13	8	5	12	7	7	7	73
2	Student performance and negative emotions	9	8	4	3	12	9	7	6	58
3	Student emotional factors	9	7	1	2	2	4	6	2	33
4	Work experience and student performance	20	10	4	4	2	5	3	4	52
5	Student biological factor and academics	11	5	6	2	3	2	5	10	44
6	Student academic achievements prediction	11	7	8	6	3	5	1	6	47
7	Student frustration	4	2	5	2	2	2	3	7	27
8	Student performance and frustration	3	7	7	6	4	3	4	7	41
9	Student frustration severity	5	7	9	5	0	8	13	13	60
10	At-risk student prediction	6	9	6	11	11	7	7	11	68
11	At-risk student cognitive skills	3	5	3	8	4	3	8	2	36
12	Cognitive skills prediction	8	6	4	7	4	5	10	10	54
13	Emotional impact on student performance	6	9	7	5	2	9	7	5	50
14	Family impact on student achievements	7	2	5	9	8	4	11	5	51
15	Student anxiety	9	7	2	11	6	7	2	5	49
16	Student stress	2	9	8	11	9	7	2	7	55
17	Review on student performance	3	13	8	3	4	5	4	3	43
18	Student performance quantization	8	3	6	2	11	2	8	4	44
19	COVID-19, frustration, and student performance	7	5	5	3	9	8	11	4	52
20	COVID-19 and at-risk student	8	8	8	4	6	3	6	2	45
21	Impact of online classes	3	2	8	8	14	7	3	2	47
22	Online classes and student learning	7	11	2	6	9	8	10	8	61
23	Learning prediction	2	3	4	4	8	2	8	5	36
24	Student learning outcome prediction	6	5	5	4	9	6	6	9	50
25	Student performance measurement	2	12	4	4	8	4	9	7	50
26	Performance measurement algorithm	5	4	2	4	9	6	6	5	41
27	Performance prediction algorithm	11	4	5	6	6	2	9	11	54
28	Student performance evaluation algorithm	7	2	6	2	6	2	3	6	34
29	Student performance prediction algorithm	12	3	9	4	8	12	15	5	68
30	Student performance measurement algorithm	3	6	2	3	7	5	2	4	32
31	Cognitive skills prediction algorithm	3	7	5	7	4	5	6	5	42

**Table 2:** Research domain-wise keyword searching results and evaluation.

	New methods	Modification	Data analysis	Psychological findings	Comparison	Analysis of application	Review work	Total number of publications
IEEE	15	23	68	65	24	10	9	214
ACM	14	16	65	70	13	14	9	201
Springer	11	17	87	32	4	7	8	166
MDPI	18	12	48	61	3	6	13	161
Hindawi	16	22	72	45	17	12	18	202
Elsevier	22	15	36	70	4	2	15	164
Wiley	8	9	66	32	14	56	17	202
Others	5	3	37	87	13	39	3	187

**Table 3:** Research domain-wise and factors-wise evaluation.

Attributes	New methods	Modification	Data analysis	Psychological findings	Comparison	Analysis of application	Review work	Total number of publications
Frustration	3	4	26	24	19	24	31	131
Frustration severity	0	0	13	33	4	35	14	99
Stress	2	2	8	19	13	19	16	79
Stress severity	0	0	18	18	9	11	21	77
Anxiety	19	12	14	17	9	14	6	91
Anxiety severity	0	2	10	22	21	14	24	93
Depression	0	0	12	20	23	12	21	88
Parents' influence	4	3	13	11	6	25	16	78
Distance from home and school	3	3	18	13	13	13	19	82
Mobile game	2	2	27	25	17	12	24	109
Outdoor game	0	0	19	7	20	13	7	66
Indoor game	0	0	22	17	21	10	10	80
Watching TV	0	0	13	10	24	18	16	81
Students social network	8	12	17	19	7	24	8	95
Gender	3	3	10	14	15	15	16	76
Parents cohabitation status	0	0	11	6	15	18	14	64
Parent service	2	4	5	15	11	3	20	60
International students	0	0	5	10	11	7	15	48

**Table 4:** Intensity of various domain contributions.

Research outcomes	New methods	Modification	Data analysis	Psychological findings	Comparison	Analysis of application	Review work	Total number of publications
SRI		3			2			5
PSP-PRP	5						4	9
AS-EDM			3					3
MAR-LD			4			6		10
RFA		6		4				10
MAR-KD			7					7
AF-TM							1	1
PSP-GPA	6			1			1	8
PSP-MC			5	5				10
EDU-DMCR		4	5					9
UPSP-EDU			6					6
PSP-OCS		7						7
PC-SD			5		5			10
EDU-DMA					3			3
EDU-DMLA						6		6
SEDU-DM			3					3
EDU-DMA			5					5
SRCT						7	3	10
PSBS	1	3					2	6
EITE		5						5
ICAP						7		7
TEO					5			5
LAD		5						5
IEDU-PM			6					6
SAFP		5				2	3	10
SPIE				9				9
PSS-TEDC			7				2	9
DMA-SD		4			2			6
EDU-DMPC		4					3	7
PSP-CA			6					6
PSS-CF	2				3		2	7
DMM-SC		1				4		5
PMSA				8				8
QACL	1		2	3			1	7
HSE				7				7
PPM-AS	2	3						5
RAE			8					8
ICASP			2		7			9
PSP-TDF	1	3				2		6
SSC					4			4
EDPLC					5			5
KPS-EDU		5				3		5
PSP-NBT	1	2						6
SMA		2					5	7
SMCS	1	1						2
PSP-LMS	5	4				5		14
TLS			2			5		7
ARICM			2					2
PSP-ALA		5						5
SARL		3					5	8
EDM		2				2		4
TRI-PAP			3		2			5
PSP-PA		2						2
AWP							3	3
TSBCP		3						3
AUD			5					5
CPU					3		2	5
DDC	1					3		4
EDMD-APS			2					2
EDM-PAAP	3		2			7	2	14
HGS-AFS			3					3
RHS			6					6
SE-TE				11				11
DAS-AARM					5			5
MPAP		4						4
EPMSS	2				5			7
LA		6						6
DLA				8			2	10
SGP-NN			3				2	5
PAP	1		2	1				4
MTQ				5				5
AD-SLS		3	3					6
CMPL	1		4					5
USWT						6		6
LAP			5					5
PSP				2				2
NSP-KDHED						2		2
MLM		1						1
ID-CS					4			4
SAP			5					5
PP-PSP					3			3
PSP-M							8	8
PA-PS	2							2
PF			2				3	5
IDF-SAP				4				4
SC-NF			6				3	9
OEP-TRF	1					7	2	10
PCS		2						2
SVA-PC				2			3	5
PSL						4		4
SAP-EDC				5				5
PRD-AP			2					2
SSP-CL	3							3
QE-ELC					5			5
GRP-OEWB							3	3
SP-DMC				2				2
PPRD-DT						4		4
SUR-MSR						4		4
SPRD-ARMBA				2				2
EXP-HPF	3							3
IPT-SP		3					4	7
DM-ETSP				7				7
EDM-ASAP				2				2
SPP-DL	2							2
DM-E		1				7	3	11
PSM-HOU	3							3
PSP-ML			3					3
PA-EDM					2			2
LS-PRDE	2		4					6
PRD-AP						2		2
HSC-SA	1		3	3			7	14
PRI-MPP			7					7
SE-OES	1		2	3			1	7
FCD-EP				7				7
EB-PSP	2	3						5
MSM-ENB			8					8
M-KME			2		7			9
EPRD-BL							5	5
MA-TE					2			2
PRD-SF-GP				2				2
SE-UT					1		6	7
SE-DRVPU				3				3
PRD-DFA	3				3			6
AANL-PISA		4						4
INT-INF							4	4
PRD-GR				4		2		6
ESEG-AP		2			1	5		8
SAG-EDM	2							2
MSD-PRD		1					3	4
PRI-SRMA	3							3
SRS-AL		3		4		4		11
SET-IFP				6				6
SRL-HYPM							2	2
SAP-DM	4			4				8
LAS-TEL		1					3	4
DMKMS		2			6			8
DSS-LE						5		5
FGCAC				2				2
SAPM	2							2
DM-PSP						2		2
OPCA	1							1
HESSP-PP		3						3
IOMC			2					2
DM-CRTL						2		2
DM-ED				5				5
FGSK-SP	2							2
EDM-ARW			3			6		9
GP-SSM	1							1
FENTP				3				3
TE-LMSF		3				5		8
RGTE							3	3
DOF-DTT	1							1
P-CSI				3				3
DTDM	1	1						2
PSP-SDMA	5	4				2		11
SAS					6			6
ODF-AFQP			2					2
PSP-OLDF		1						1
EDM-S		3					5	8
EDM-RSA		1				2		3
ASP-DCBC			4		2			6
CSP-LCV		2						2
EEDM-IPC	2							2
PSP-C				3	3			6
PSP-DMT	2					8		10
WUGC						2		2
SEDM-PSP							4	4
LAEDM-CC						2		2
PSP-EDT			3			5		8
TQSA-ES	4					4		8
PMTP							3	3
DFUS				3				3
SPP-CS	1					2		3
MED-CS		4						4
IAPP	1				4			5
MM-SN						7		7
TQ-CS		5		3				8
MA-FTE	2		2					4
FGAM						7		7
MRF-CA	2		3					5
GSM	3			2			3	3
OCM							3	3
LRMP				4				4
IDK		7			4			11
EDM				2				2
ILA-EDM							5	5
RPP	1		4			2		7
SP-RBFNN&PCA			4					4
SP-MLR&PCA	4					5		9
WTM			1		6			7
SCS		4						4
LF-PP				2				2
SA						5		5
SET	4				4			8
WBLC		1		2				3
MRC						3		3
MBA-GL							2	2
SPM			4					4
S-GPA				5		1		6
PFDM							6	6
DMT-SN		3						3
PPS-COVID-19				4				4
ATI-F		2						2
NA-FD-COVID-19					4		3	7
COVID-19-AS	2							2
PI-COVID-19			2					2
SD-COVID-19					2			2
Edu-COVID-19			2					2
NCAS-COVID-19							2	2
Imp-COVID-19			2	3				5
SS-PPP-DM	1					8		9
A-EDM-TD			3					3
RPSP-DMT							3	3
SPP-CL						6		6
ER-KCP		3						3
SDP				11				11
EDP-DM				4				4
PAP-SH	7							7
HMRS		2						2
IGR-PSP		3						3
PSPP-ML						2		2
ECE-RL	3		3	2			2	10
SML-OC							2	2
Inf-COVID-19					3			3
PEEP-COVID-19			2			5		7
ATI-F		4					5	9
CCI-OC					4			4
ETES-COVID-19		3		2				5
TFL-SF						4		4
OC-BL		6						6
NP-PSP						5		5
SPP-BL			2					2
RSNL				6				6
DN-CS	1		3					4
PR-MS				7				7
DSF-HB	1		2	3			6	12
VFP-C				7				7
EAK-P	2	3						5
SP-EG-MM			8					8
LMS-CAP			2		7			9
FDG	2							2
BFE				3				3
AD-CS	2	3						5
SP-ALA			8					8
TVL-CA			2		7			9
EAG-CSC				4				4
ML-CSC			2					2
SP-DM-LAT	4		5					9
S-GC			4					4
MR-PCQ				3				3
MPA-M	1					2		3
MCA-E		4					3	7
T-PR				3				3
NS-SE						5		5
ARFE			3					3
CSMA				8				8
NT-PPCS	3							3
MSG-IC					5			5
GD-ATC		3						3
GD-AT-SCI			3					3
LS-ESP-R			4					4
GD-SE			2					2
GD-AT-IT				4				4
GD-RC		3				2		5
GD-LTS			2					2
SG-TM-CAP	2					4		6
GDSL			4					4
GD-MS-SL		3					5	8
GD-MR				5				5
DSS-CP	2	6				2		10
GD-TET			4					4
GD-HSS			2		3			5
TP-MA				4			3	7
GD-NCS	1		2					3
GD-SP-EC				4				4
SSG		4						4
GD-DSS			3			5		8
ETP-SSA				5				5
GES-E			3					3
PSD		2						2
IQ-PAP					4			4
TSI-SSC							3	3
BFP-MA		3					4	7
ESF-SS				2				2
FPP-AUS			5	6				11
ACA			3				6	9
AAGT	1	3		5				9
SLC-A		2					3	5
RHAS				8				8
PSO-LPS	1	2						3
								1497

**Table 5:** Abbreviation and acronym.

Abbreviation	Acronym
SRI	The dimensionality of student ratings of instruction: what we know and what we do not
PSP-PRP	Predicting student performance on post-requisite skills using prerequisite
AS-EDM	An approachable analytical study on big educational data mining
MAR-LD	Mining association rules between sets of items in large databases
RFA	Clarify of the random forest algorithm in an educational field
MAR-KD	Knowledge discovery from academic data using association rule mining
AF-TM	How automated feedback through text mining changes plagiaristic behavior in online assignments
PSP-GPA	Predicting students final GPA using decision trees
PSP-MC	Analyzing students performance using multicriteria classification
EDU-DMCR	Data mining in educational technology classroom research
UPSP-EDU	Analyzing undergraduate students' performance using educational data mining
PSP-OCS	Student performance predicition and optimal course selection
PC-SD	Probabilistic classifiers and statistical dependency
EDU-DMA	Educational data mining: an advance for intelligent systems in education
EDU-DMLA	Educational data mining and learning analytics
SEDU-DM	The state of educational data mining in 2009
EDU-DMA	Educational data mining applications and tasks
SRCT	Student ratings of college teaching
PSBS	Predicting drop-out from social behavior of students
EITE	Ensemble learning for estimating individualized treatment effects in student success studies
ICAP	Identifying the comparative academic performance of secondary schools
TEO	Taxonomy of educational objectives
LAD	The design, development, and implementation of student-facing learning analytics dashboards
IEDU-PM	Clustering for improving educational process mining
SAFP	Determining students' academic failure profile founded on data mining methods
SPIE	Student perceptions and instructional evaluations
PSS-TEDU	Predicting student success using data generated in traditional educational environments
DMA-SD	Data mining application on students' data
EDU-DMPC	Educational data mining for prediction and classification of engineering students achievement
PSP-CA	A comparative analysis of techniques for predicting student performance
PSS-CF	Predicting students success in courses via collaborative filtering
DMM-SC	Data mining models for student careers
PMSA	Blending measures of programming and social behavior into predictive models of students achievement in early computing courses
QACL	Quantitative approach to collaborative learning
HSE	Will teachers receive higher student evaluations by giving higher grades and less course work?
PPM-AS	Student performance prediction model for early-identification of at-risk students in traditional classroom settings
RAE	Regression analysis by example
ICASP	Mining the impact of course assignments on student performance
PSP-TDF	Predicting student performance in an ITS using task-driven features
SSC	Soft subspace clustering of categorical data with probabilistic distance
EDPLC	Early detection prediction of learning outcomes in online short-courses via learning behaviors
KPS-EDU	Tracking knowledge proficiency of students with educational priors
PSP-NBT	Exploration of classification using NB tree for predicting students' performance
SMA	Student modeling approaches: a literature review for the last decade
SMCS	An ontological approach for semantic modeling of curriculum and syllabus in higher education
PSP-LMS	Predicting student performance from LMS data
TLR	Organizing knowledge syntheses: a taxonomy of literature reviews
ARICM	Analysis of academic results for informatics course improvement using association rule mining
PSP-ALA	Predicting student performance using advanced learning analytics
SARL	Seeding the survey and analysis of research literature with text mining
EDM	A systematic review of educational data mining
TRI-PAP	Do the timeliness, regularity, and intensity of online work habits predict academic performance?
PSP-PA	Predicting student performance using personalized analytics
AWP	Automated analysis of aspects of written argumentation
TSBCP	Predicting performance form test scores using back propagation and counter propagation
AUD	The text mining handbook: advanced approaches in analyzing unstructured data,cambridge
CPU	Cell phone usage and academic performance
DDC	Learning analytics: drives, developments and challenges
EDMD-APS	Educational data mining discovery standards of academic performance by students
EDM-PAAP	Educational data mining: predictive analysis of academic performance
HGS-AFS	Do high grading standards affect student performance?
RHS	Retrieving hierarchical syllabus items for exam question analysis
SE-TE	Are student evaluations of teaching effectiveness valid for measuring student learning outcomes in business related classes?
DAS-AARM	Drawbacks and solutions of applying association rule mining in learning management systems
MPAP	Model prediction of academic performance for first year students
EPMSS	Evaluating predictive models of student success: closing the methodological gap
LA	Learning analytics should not promote one size fits all
DLA	Detecting learning strategies with analytics: links with self-reported measures and academic performance
SGP-NN	Explaining student grades predicted by a neural network
PAP	Predicting academic performance
MTQ	Measuring teaching quality in higher education
AD-SLS	Towards automatically detecting whether student learning is shallow
CMPL	An application of classification models to predict learner progression in tertiary education
USWT	Utilizing semantic web technologies and data mining techniques to analyze students learning and predict final performance
LAP	A model to predict low academic performance at a specific enrollment using data mining
PSP	Predicting students performance in educational data mining
NSP-KDHED	A new student performance analysing system using knowledge discovery in higher educational databases.
MLM	Comparison of machine learning methods for intelligent tutoring systems
ID-CS	Individual differences related to college students' course performance in calculus ‖
SAP	Student academic performance prediction by using a decision tree algorithm.
PP-PSP	Performance prediction based on particle swarm optimization
PSP-M	Poverty and student performance in Malaysia
PA-PS	Physical activity is not related to performance at school
PF	The power of feedback, review of educational research
IDF-SAP	Identifying key factors of student academic performance by subgroup discovery
SC-NF	Student classification for academic performance prediction using neuro fuzzy in a conventional classroom
OEP-TRF	Online education performance predication via time-related features
PCS	Programming content semantics: an evaluation of visual analytics approach
SVA-PC	Semantic visual analytics for today's programming courses
PSL	A systematic review of studies on predicting student learning outcomes using analytics
SAP-EDC	Predicting student academic performance in an engineering dynamics course: a comparison of four types of predictive mathematical models
PRD-AP	Predicting student's academic performance: comparing artificial neural network, decision tree, and linear regression
SSP-CL	Analyzing student spatial deployment in a computer laboratory
QE-ELC	Quality enhancement for e-learning courses: the role of student feedback
GRP-OEWB	Improving accuracy of students' final grade prediction model using optimal equal width binning and synthetic minority over-sampling technique
SP-DMC	Student performance prediction by using data mining classification algorithms
PPRD-DT	Performance prediction of engineering students using decision trees
SUR-MSR	A survey and taxonomy of approaches for mining software repositories in the context of software evolution
SPRD-ARMBA	A review and performance prediction of students' using an association rule mining based approach
EXP-HPF	Exploring the high potential factors that affects students' academic performance
IPT-SP	Analysing the impact of poor teaching on student performance
DM-ETSP	Data mining based analysis to explore the effect of teaching on student performance
SPP-DL	Gritnet: student performance prediction with deep learning
DM-E	Data mining and education
PSM-HOU	Predicting students marks in hellenic open university
PSP-ML	Predicting postgraduate students' performance using machine learning techniques
PA-EDM	Review on prediction algorithms in educational data mining
LS-PRDE	Literature survey on student's performance prediction in education using data mining techniques
PRD-AP	Predicting student academic performance
HSC-SA	Online self-paced high-school class size and student achievement
PRI-MPP	Predictor relative importance and matching regression parameters
SE-OES	Finding similar exercises in online education systems
FCD-EP	Fuzzy cognitive diagnosis for modeling examine performance
EB-PSP	An ensemble-based semi-supervised approach for predicting students' performance
MSM-ENB	Measuring the (dis-) similarity between expert and novice behaviors as serious games analytics
M-KME	Mining for topics to suggest knowledge model extensions
EPRD-BL	Applying learning analytics for the early prediction of students' academic performance in blended learning
MA-TE	Whose feedback? A multilevel analysis of student completion of end-of-term teaching evaluations
PRD-SF-GP	Predicting student failure at school using genetic programming and different data mining approaches with high dimensional and imbalanced data
SE-UT	Students' evaluations of university teaching: Dimensionality, reliability, validity, potential biases and usefulness
SE-DRVPU	Students' evaluations of university teaching: Dimensionality, reliability, validity, potential biases and usefulness
PRD-DFA	Predicting student outcomes using discriminant function analysis
AANL-PISA	An overview of using academic analytics to predict and improve students' achievement: a proposed proactive intelligent intervention
INT-INF	Constructing interpretive inferences about literary text: the role of domain-specific knowledge
PRD-GR	Predicting grades
ESEG-AP	Early segmentation of students according to their academic performance: a predictive modeling approach
SAG-EDM	A framework for smart academic guidance using educational data mining
MSD-PRD	Mining students' data for prediction performance
PRI-SRMA	Preferred reporting items for systematic reviews and meta-analyses: the PRISMA statement
SRS-AL	A semantic recommender system for adaptive learning
SET-IFP	Students evaluating teachers: exploring the importance of faculty reaction to feedback on teaching
SRL-HYPM	Self-regulated learning with hypermedia: the role of prior domain knowledge
SAP-DM	Modeling and predicting students' academic performance using data mining techniques
LAS-TEL	Lexical analysis of syllabi in the area of technology enhanced learning
DMKMS	Student data mining solution-knowledge management system
DSS-LE	Decoding student satisfaction: how to manage and improve laboratory experience
FGCAC	Student ability best predicts final grade in a college algebra course
SAPM	Student academic performance monitoring and evaluation
DM-PSP	Data mining approach for predicting student performance
OPCA	Optimizing partial credit algorithms
HESSP-PP	Is alcohol affecting higher education students' performance: searching and predicting pattern
IOMC	Towards the integration of multiple classifier pertaining to the student's performance prediction
DM-CRTL	A data mining view on classroom teaching language
DM-ED	Application of data mining in educational databases for predicting academic trends and patterns
FGSK-SP	Using fine-grained skill models to fit student performance
EDM-ARW	Educational data mining: a survey and a data mining-based analysis of recent works
GP-SSM	Grade prediction with course and student specific models
FENTP	Feature extraction for next-term prediction of poor student performance
TE-LMSF	Teaching evaluation using data mining on moodle LMS forum
RGTE	The role of gender in students' ratings of teaching quality in computer science and environmental engineering
DOF-DTT	Drop out feature of student data for academic performance using decision tree techniques
P-CSI	Programming: predicting student success early in CSI
DTDM	Decision trees and decision-making
PSP-SDMA	Predicting student performance: a statistical and data mining approach
SAS	A sentiment analysis system to improve teaching and learning
ODF-AFQP	Ontology driven framework for assessing the syllabus fairness of a question paper
PSP-OLDF	Predicting students' final performance from participation in on-line discussion forums
EDM-S	Educational data mining: a survey from 1995 to 2005
EDM-RSA	Educational data mining: a review of the state of the art
ASP-DCBC	Analyzing student performance using sparse data of core bachelor courses
CSP-LCV	Centralized student performance prediction in large courses based on low-cost variables in an institutional context
EEDM-IPC	Evaluating the effectiveness of educational data mining techniques for early prediction of students' academic failure in introductory programming courses
PSP-C	Prediction of students' academic performance using clustering
PSP-DMT	A review on predicting students' performance using data mining techniques
WUGC	Web-based undergraduate chemistry problem-solving: the interplay of task performance, domain knowledge and web-searching strategies
SEDM-PSP	A survey on various aspects of education data mining in predicting student performance
LAEDM-CC	Learning analytics and educational data mining: towards communication and collaboration
PSP-EDT	Predictive modeling of students performance through the enhanced decision tree
TQSA-ES	What is the relationship between teacher quality and student achievement? An expletory study
PMTP	A predictive model for standardized test performance in Michigan schools
DFUS	Determination of factors influencing the achievement of the first-year university students
SPP-CS	Next-terms student performance prediction: a case study
MED-CS	Mining educational data to improve students' performance: a case study
IAPP	Improving academic performance prediction by dealing with class imbalance
MM-SN	Proposing stochastic probability-based math model and algorithms utilizing social networking and academic data
TQ-CS	Teaching quality matters in higher education: a case study
MA-FTE	Meta-analysis of faculty's teaching effectiveness: student evaluation of teaching ratings and student learning
FGAM	Analysis of the impact of action order on future performance: the fine-grain action model
MRF-CA	Map-reduce framework based cluster architecture for academic students' performance prediction
GSM	Google Scholar coverage of a multidisciplinary field
OCM	The opportunity count model: a flexible approach to modeling student performance
LRMP	Predicting students' performance in final examination using linear regression and multilayer perceptron
IDK	Fast searching for information on the internet to use in a learning context: the impact of domain knowledge
EDM	Educational data mining acceptance among undergraduate students
ILA-EDM	Participation-based student final performance prediction model through interpretable genetic programming: integrating learning analytics, educational data mining and theory
RPP	Improving retention performance prediction with prerequisite skill features
SP-RBFNN&PCA	Predicting honors student performance using RBFNN and PCA method
SP-MLR&PCA	Predicting students' academic performance using multiple linear regression and principal component analysis
WTM	Web-based collaborative writing in L2 contexts: methodological insights from text mining
SCS	Chinese undergraduates' perceptions of teaching quality and the effects on approaches to studying and course satisfaction
LF-PP	Can online discussion participation predict group project performance? Investigating the roles of linguistic features and participation patterns
SA	Improving early prediction of academic failure using sentiment analysis on self-evaluated comments
SET	The use and misuse of student evaluations of teaching
WBLC	A multivariate approach to predicting student outcomes in web-enabled blended learning courses
MRC	Mendeley: creating communities of scholarly inquiry through research collaboration
MBA-GL	A model-based approach to predicting graduate-level performance using indicators of undergraduate-level performance
SPM	Students performance modeling based on behavior pattern
S-GPA	Predicting students' GPA and developing intervention strategies based on self-regulatory learning behaviors
PFDM	Towards parameter-free data mining: mining ‘educational data with yacaree
DMT-SN	A survey of data mining techniques for social network analysis
PPS-COVID19	New realities for polish primary school informatics education affected by COVID-19
ATI-F	Affect-targeted interviews for understanding student frustration
NA-FD-COVID19	Unhappy or unsatisfied: distinguishing the role of negative affect and need frustration in depressive symptoms over the academic year and during the COVID-19 pandemic
COVID19-AS	COVID-19 disruption on college students: academic and socioemotional implications
PI-COVID19	The psychological impact of COVID-19 on the mental health of the general population
SD-COVID19	Social distancing in covid-19: what are the mental health implications?
Edu-COVID19	Education and the COVID-19 pandemic
NCAS-COVID19	Negative emotions, cognitive load, acceptance, and self-perceived learning outcome in emergency remote education during COVID-19
Imp-COVID19	The impact of COVID-19 on education insights from education at a glance 2020
SS-PPP-DM	Study on student performance estimation, student progress analysis, and student potential prediction based on data mining
A-EDM-TD	Application of educational data mining approach for student academic performance prediction using progressive temporal data
RPSP-DMT	A review on predicting students' performance using data mining techniques
SPP-CL	Student performance analysis and prediction in classroom learning: a review of educational data mining studies
ER-KCP	Exercise recommendation based on knowledge concept prediction
SDP	Student dropout prediction
EDP-DM	Early dropout prediction using data mining: a case study with high school students
PAP-SH	Predicting academic performance by considering student heterogeneity
HMRS	Helping university students to choose elective courses by using a hybrid multicriteria recommendation system with genetic optimization
IGR-PSP	Inductive Gaussian representation of user-specific information for personalized stress-level prediction
PSPP-ML	Pre-course student performance prediction with multi-instance multi-label learning
ECE-RL	What students want? Experiences, challenges, and engagement during emergency remote learning amidst COVID-19 crisis
SML-OC	A survey of machine learning approaches for student dropout prediction in online courses
Inf-COVID19	Covid-19 and student performance, equity, and us education policy: lessons from pre-pandemic research to inform relief, recovery, and rebuilding
PEEP-COVID19	COVID19 and student performance equity, and us education Policy: Lessons from pre-pandemic research to inform relief, recovery, and rebuliding.
ATI-F	“Affect-targeted interviews for understanding student frustration”, in international conference on artificial intelligence in education
CCI-OC	Common challenges for instructors in large online course: strategies to mitigate student and instructor frustration
ETES-COVID19	Effective teaching and examination strategies for undergraduate learning during COVID-19 school restrictions
TFL-SF	Teacher feedback literacy and its interplay with student feedback literacy
OC-BL	Challenges in the online component of blended learning: a systematic review
NP-PSP	Feature extraction for next-term prediction of poor student performance
SPP-BL	Student performance prediction based on blended learning
RSNL	Robust student network learning
DN-CS	Deep network for the iterative estimations of students' cognitive skills
PR-MS	Parents' role in the academic motivation of students with gifts and talents
DSF-HB	Detecting student frustration based on handwriting behavior
VFP-C	The validity of a frustration paradigm to assess the effect of frustration on cognitive control in school-age children
EAK-P	Ekt: exercise-aware knowledge tracing for student performance prediction
SP-EG-MM	Predicting student performance in an educational game using a hidden Markov model
LMS-CAP	Massive lms log data analysis for the early prediction of course-agnostic student performance
FDG	Frustration drives me to grow
BFE	Between frustration and education: transitioning students' stress and coping through the lens of semiotic cultural psychology
AD-CS	Automatic discovery of cognitive skills to improve the prediction of student learning
SP-ALA	Predicting student performance using advanced learning analytics
TVL-CA	Time-varying learning and content analytics via sparse factor analysis
EAG-CSC	Emotions, age, and gender based cognitive skills calculations
ML-CSC	Machine learning based cognitive skills calculations for different emotional conditions
SP-DM-LAT	Predicting student performance using data mining and learning analytics techniques: a systematic literature review
S-GC	Should I grade or should I comment: links among feedback, emotions, and performance
MR-PCQ	Modeling the relationship between students' prior knowledge, causal reasoning processes, and quality of causal maps
MPA-M	A multilayer prediction approach for the student cognitive skills measurement
MCA-E	A meta-cognitive architecture for planning in uncertain environments
T-PR	The influence of teacher and peer relationships on students
NS-SE	National Society for the Study of Education
ARFE	Automatically recognizing facial expression: predicting engagement and frustration
CSMA	A biologically inspired cognitive skills measurement approach
NT-PPCS	A novel technique for the evaluation of posterior probabilities of student cognitive skills
MSG-IC	Medical student gender and issues of confidence
GD-ATC	Gender differences in student attitudes toward computers
GD-AT-SCI	Gender differences in student attitudes toward science: a meta-analysis of the literature from 1970 to 1991
LS-ESP-R	A longitudinal study of engineering student performance and retention III. Gender differences in student performance and attitudes
GD-SE	Gender differences in student ethics: Are females really more ethical? Gender differences in teacher-student interactions in science classrooms
GD-AT-IT	Gender differences in attitudes towards information technology among Malaysian student teachers: a case study at University Putra Malaysia
GD-RC	Gender differences in the response to competition
GD-LTS	Gender differences in the learning and teaching of surgery: a literature review
SG-TM-CAP	Student gender and teaching methods as sources of variability in children's computational arithmetic performance
GDSL	Gender difference and student learning
GD-MS-SL	Gender difference in student motivation and self-regulation in science learning: a multigroup structural equation modeling analysis
GD-MR	Gender differences in the influence of faculty-student mentoring relationships on satisfaction with college among African-Americans
DSS-CP	Differences of students' satisfaction with college professors: the impact of student gender on satisfaction
GD-TET	Gender differences in teachers' perceptions of students' temperament, educational competence, and teachability
GD-HSS	Gender differences in factors affecting academic performance of high school students
TP-MA	Influence of elementary student gender on teachers' perceptions of mathematics achievement
GD-NCS	Gender differences in alcohol-related non-consensual sex, cross-sectional analysis of a student population
GD-SP-EC	Gender differences in students' and parents' evaluative criteria when selecting a college
SSG	Social influences, school motivation, and gender differences: an application of the expectancy-value theory
GD-DSS	Gender differences in the dimensionality of social support
ETP-SSA	Early teacher perceptions and later student academic achievement
GES-E	Gender, ethnicity, and social cognitive factors predicting the academic achievement of students in engineering
PSD	Predicting students drop out: a case study
IQ-PAP	Self-discipline outdoes IQ in predicting academic performance of adolescents
TSI-SSC	Observations of effective teacher-student interactions in secondary school classrooms: predicting student achievement with the classrooms assessment scoring system-secondary
BFP-MA	Role of the big five personality traits in predicting college students' academic motivation and achievement
ESF-SS	Using emotional and social factors to predict student success
FPP-AUS	Who succeeds at university? Factors predicting academic performance in first-year Australian university students
ACA	Predicting academic achievement with cognitive ability
AAGT	Advancing achievement goal theory: using goal structures and goal orientations to predict students' motivation, cognition, and achievement
SLC-A	Short-term and long-term consequences of achievement goals: predicting interest and performance over time
RHAS	Role of hope in academic and sports achievement
PSO-LPS	Prediction of school outcomes based on early language production and socioeconomic factors

**Table 6:** Summary of potential research challenges and recommendation.

S.No	Research question	Remarks	Recommendations
1	What are the applications of student performance prediction systems?	Prediction of at-risk students for special treatment and counseling sessions.	Mathematically model emotional attributes, family issues, study schedules, and institutional attributes all together to develop a significant prediction system.
		If students cannot achieve an excellent academic score, then the performance prediction system assists students in observing the main reason behind the low performance.	If the prediction system considers a large number of influential factors, then the academic achievement of excellent can also be advanced.
		Advance students' academic achievements.	Modulates the relationship between behavior and students' performance
		Monitor students' behavior such as interaction and attitude towards teacher, seriousness, and unseriousness in the classroom	
2	What are the factors that can optimize student performance prediction?	They include but are not limited to family-related factors, emotional factors, gender description, and institution-related factors.	Initiate pilot projects with an assumption-based dataset. The assumptions should be based on earlier studies of psychology, data analysis, and data mining.
		Emotional factors, such as frustration, anxiety, stress, and depression.	Analyze the performance of at-risk students while mathematically modeling the association among students' emotional, family, and institution-related attributes.
		Quantize family factors, i.e., parents' positive and negative roles, including overexpectation of parents and positive involvement of parents in children's daily cognitive activities.	Perform factorization of gender because earlier studies depict that gender difference magnitude is sometimes dependent on other factors such as cultures, socioeconomic condition, language, age, etc.
		Literature studies are evidenced with many contributions to gender differences. They show that different gender individuals perform differently during cognitive activities, solving assignments, attempting quizzes, and examinations studies.	Explore instructor teaching methodology, interaction with a student advisor, extra curriculum activities in the institution, student complaint platform, the distance between the institution and students' residence, transport facility, and the behavior of the friends.
		Different institutional factors directly or indirectly influence students' performance.	
3	What is the intensity of research findings in the field of student performance prediction systems optimization?	Intensity of psychological findings	These findings are not synchronized and linked toward a significant student performance prediction model.
		Intensity of data analysis findings	So, the main challenge is to provide an effective platform where future researchers can collaborate and synchronize the prior findings. Also, pilot projects based on the assumption-based dataset are highly recommended. Successful pilot project implementation will pave the way for quick optimization of existing systems.
		Intensity of students' performance prediction systems	
4	Are the findings of psychological studies, data mining, and contribution in algorithms synchronized with each other for the viability of the pilot project?	The intensity of publications contributing to student performance prediction is quite good, but these contributions are not synchronized with each other.	Mathematically model emotional, family, and institution-related attributes.
5	How do synchronization and coordination of prior psychological, data mining, and algorithmic findings contribute to the effective educational system via student performance prediction algorithm?	Every part of the student performance prediction area of research is interlinked. The psychological result verifies the emotional change during the evaluations of the frustration, severity, anxiety, and stress. The data analysis findings associate the student attributes. The student performance prediction algorithm mathematical model the statistical association among the student influencing factors and their performance outcome.	If these findings are linked with the objective of qualitative data repositories and algorithms, then, we can move toward an efficient student performance prediction system.

## Data Availability

The screening data are available from the corresponding author, upon reasonable request.
